# Improving Kinetics of “Click-Crosslinking” for Self-Healing Nanocomposites by Graphene-Supported Cu-Nanoparticles

**DOI:** 10.3390/polym10010017

**Published:** 2017-12-24

**Authors:** Neda Kargarfard, Norman Diedrich, Harald Rupp, Diana Döhler, Wolfgang H. Binder

**Affiliations:** 1 Faculty of Natural Science II, Martin Luther University Halle-Wittenberg, Von-Danckelmann-Platz 4, D-06120 Halle (Saale), Germany; kargarfard@ipfdd.de (N.K.); norman.diedrich@student.uni-halle.de (N.D.); harald.rupp@chemie.uni-halle.de (H.R.); 2 Leibniz-Institut für Polymerforschung Dresden e. V., Abteilung Reaktive Verarbeitung, Hohe Str. 6, D-01069 Dresden, Germany

**Keywords:** TRGO, copper nanoparticles, CuAAC crosslinking, self-healing nanocomposite

## Abstract

Investigation of the curing kinetics of crosslinking reactions and the development of optimized catalyst systems is of importance for the preparation of self-healing nanocomposites, able to significantly extend their service lifetimes. Here we study different modified low molecular weight multivalent azides for a capsule-based self-healing approach, where self-healing is mediated by graphene-supported copper-nanoparticles, able to trigger “click”-based crosslinking of trivalent azides and alkynes. When monitoring the reaction kinetics of the curing reaction via reactive dynamic scanning calorimetry (DSC), it was found that the “click-crosslinking” reactivity decreased with increasing chain length of the according azide. Additionally, we could show a remarkable “click” reactivity already at 0 °C, highlighting the potential of click-based self-healing approaches. Furthermore, we varied the reaction temperature during the preparation of our tailor-made graphene-based copper(I) catalyst to further optimize its catalytic activity. With the most active catalyst prepared at 700 °C and the optimized set-up of reactants on hand, we prepared capsule-based self-healing epoxy nanocomposites.

## 1. Introduction

Self-healing approaches do have a significant potential in polymeric materials, especially those based on embedded capsule systems [1]. The molecular design of such self-healing materials requires fast and efficient crosslinking processes, which often are afforded by catalytic reactions using homogeneous and heterogeneous chemistry [2]. In the past, a plethora of such processes has been reported, based on e.g., ring-opening metathesis polymerization (ROMP)- (….) [1,3,4,5,6,7,8,9,10], copper(I)-catalyzed azide-alkyne cycloaddition (CuAAC)- (….) [11,12,13,14,15,16,17,18,19,20,21,22,23,24,25,26,27], isocyanate- [28,29,30,31,32,33,34,35,36,37], thiol- [38,39,40,41,42,43,44,45,46] and hydrosilylation-chemistry [47,48], many of them using metal-catalysis.

In the field of ROMP-based self-healing [7,9], the curing behavior of renewable norbornenyl-functionalized isosorbide monomers in the copolymerization with dicyclopentadiene (DCPD) was investigated exhibiting a higher reactivity, consequently facilitating low temperature ROMP. Additionally, higher crosslinking densities were observed, resulting in improved thermal and mechanical properties highlighting the potential of renewable ROMP-monomers towards self-healing applications [9].

In particular, the search for graphene-supported catalysts [12,19,21,22,23] is ongoing, effecting Ru-based crosslinking within nanocomposites. Thus, graphene oxide (GO)-supported Grubb’s catalysts (GO-HG1 and GO-HG2) have been prepared and investigated in the ROMP of 5-ethylidene-2-norbornene at 40 to 60 °C. While showing catalytic activity the amount of catalyst was reduced from 5.0 to 0.5 wt % [7].

We recently reported on a “click”-based crosslinking chemistry useful as a principle for optimized self-healing materials [12]. In this particular system, multivalent azides and alkynes are crosslinked by the use of a Cu(I)-catalyst, acting as the known essential catalytic system for “click”-chemistry [12,49,50,51,52,53]. Of particular importance is the ability to tune the temperature at which crosslinking takes place, thus enabling self-healing at room temperature or even below [11,17]. The central principal of action is the use of encapsulated low molecular weight azides [19,22] or azide-functionalized polymers [11,14,15,17,20,27], and low molecular weight alkynes [15,19,20,22] or alkyne-functionalized polymers [11,14], embedded within capsules sized from 100 nm up to microns [13,15,19,54]. Whereas the uncatalyzed process is conventionally taking place at temperatures close to ~160 °C [55,56], the use of Cu(I) as catalyst can significantly lower the crosslinking temperature, together with an increase in crosslinking efficiency [49,50,51]. The kinetics of “click-crosslinking“ reactions was studied by us [12] and others [24,25] and the huge potential of the CuAAC towards self-healing materials is highlighted by the observation of autocatalytic effects in the melt state. Thus, the formed 1,2,3-triazole rings can act as internal ligands consequently significantly increasing the reaction rate [11], well known before for the addition of external triazole-containing ligands [57]. By additionally designing chelation assisting azides, the self-healing temperature could be tuned, and efficient network formation was even observed below room temperature (*T* = 10 °C) [17].

When probing a large variety of homogeneous and heterogeneous catalysts, the use of finely dispersed Cu_2_O on graphene-oxide (TRGO-Cu_2_O) displayed high catalytic activity in various “click-crosslinking” reactions in the melt, easy and efficient recyclability in solution experiments as well as high stability against oxygen [21]. Consequently, the catalyst was used to generate graphene-based nanocomposites via the CuAAC without addition of a base or any reducing agents. As the cheap and easy up-scalable catalyst acted as reinforcing filler, the mechanical, thermal and conductive properties of the final resin were improved [22], opening the possibility to generate self-healing capsule-based epoxy nanocomposites showing quantitative healing at room temperature within 36 h [19].

For a capsule-based self-healing approach, the encapsulation of the azides required a careful tuning of their hydrophobicity. It was unclear though, how and whether small changes in the azide-monomer would change its reactivity within the same click-system. Thus, a modification of the azide-monomer to a more hydrophobic surrounding deemed interesting, most of all to facilitate its encapsulation via emulsion-processes. Therefore, in this study we investigate the influence of small substitutions within the trivalent azides on the reaction kinetics investigated via DSC as well as in view of different homogeneous and heterogeneous copper(I) catalysts. 

## 2. Materials and Methods

### 2.1. Materials

Trimethylol propane (purity > 97%), trimethylol propane triglycidyl ether (technical grade, ^1^H-NMR spectrum in Supplementary Materials Figure S1), propargyl bromide (80 wt % solution in toluene), sodium sulphate (Na_2_SO_4_, purity > 99%), sodium azide (NaN_3_, purity 99.5%), 4-dimethylaminopyridine (DMAP, purity 99%), graphite (synthetic grade), potassium permanganate (KMnO_4_, analytical grade), copper(II) acetate hydrate (purity 98%), acetic anhydride (purity > 99%) and decanoyl chloride (purity > 98%) were purchased from Sigma Aldrich (Taufkirchen, Germany) and were used as received. Sodium hydroxide (NaOH, purity > 99%), ammonium chloride (NH_4_Cl, purity > 99%), concentrated sulphuric acid (H_2_SO_4_, 95–98%), hydrochloric acid (HCl, 37%) and *N,N*-dimethylformamide (DMF, purity > 99%) were ordered from Grüssing (Filsum, Germany) and DMF was freshly distilled from CaH_2_ under nitrogen atmosphere before use. Butyryl chloride (purity > 98%) and calcium hydride (CaH_2_, purity > 92%) were obtained from Alfa Aesar (Karlsruhe, Germany). Dichloromethane (DCM, purity > 98%) was purchased from Overlack (Mönchengladbach, Germany), chloroform (CHCl_3_, purity > 98%) was received from VWR (Darmstadt, Germany) and methanol (MeOH, purity > 99.9%) was obtained from Brenntag (Mühlheim an der Ruhr, Germany) and all solvents were distilled prior use. Tetra-*n*-butylammonium bromide (TBAB, purity > 99%) was purchased from TCI (Eschborn, Germany) and sodium chloride (NaCl, purity > 99%), *o*-phosphoric acid (H_3_PO_4_, 85%), hydroxide peroxide (H_2_O_2_, 30 wt %) were received from Carl Roth (Karlsruhe, Germany) and were used as received. The synthesis of trimethylolpropane tripropargyl ether (TMPTE, **1**) was done according to literature [22,58] and was further optimized to yield exclusively **1** (see Supplementary Materials, Scheme S1). The synthesis of (azidated trimethylolpropane triglycidyl ether, N_3_TMPTE, **2**) was done according to literature [22,59] with slight modifications (see Supplementary Materials Scheme S2).

### 2.2. Methods

Nuclear magnetic resonance (NMR) spectroscopy: All NMR spectra were recorded on a Varian Gemini 400 spectrometer from Agilent Technologies (Waldbronn, Germany) at 400 MHz at 27 °C. Deuterated chloroform (CDCl_3_, purity > 99.8%, stabilized with Ag) was used as solvent and was purchased from Chemotrade (Düsseldorf, Germany). The chemical shifts were recorded in ppm and all the coupling constants in Hz. MestRec v.4.9.9.6 (Mestrelab Research, A Coruña, Spain, 2016) was used for data interpretation.

Attenuated total reflection Fourier transformed infrared (ATR-FTIR) spectroscopy: All ATR-FTIR spectra were recorded on a Bruker Tensor VERTEX 70 from Bruker Optics GmbH (Leipzig, Germany) equipped with a heatable Golden Gate Diamond ATR plate from Specac (Orpington, Kent, UK). Opus 6.5 (Bruker Optik GmbH, Leipzig, Germany, 2008) and OriginPro 8G (Version 8.0951, OriginLab Corporation, Northampton, MA, USA, 2008) was used for data interpretation.

Thin layer chromatography (TLC): TLC was performed with TLC aluminum sheets (silica gel 60 F254) obtained from Merck (Darmstadt, Germany). Spots on the TLC plates were visualized by UV light (254 or 366 nm) or by oxidizing agents like “blue stain” consisting of Ce(SO_4_)_2_∙4H_2_O (1.0 g, analytical grade, Sigma Aldrich (Taufkirchen, Germany)), (NH_4_)_6_Mo_7_O_24_∙4H_2_O (2.5 g, analytical grade, Sigma Aldrich (Taufkirchen, Germany)), dissolved in concentrated H_2_SO_4_ (6.0 mL, 95–97%, Grüssing (Filsum, Germany)) and distilled water (90.0 mL).

Electrospray ionization time of flight mass spectrometry (ESI-TOF MS): ESI-TOF MS was performed with a micro TOF focus from Bruker Daltonics GmbH (Bremen, Germany) with an electrospray ionization source (ESI source). Samples were dissolved in CHCl_3_ (HPLC grade, VWR (Darmstadt, Germany)) or MeOH (HPLC grade, VWR (Darmstadt, Germany)) and sodium iodide (purity > 99.9%, Sigma Aldrich (Taufkirchen, Germany)), 20 mg∙mL^−1^ acetone (HPLC grade, Sigma Aldrich (Taufkirchen, Germany)) was added. The analyte was injected with a 180 µL∙h^−1^ flow rate at 180 °C.

Differential scanning calorimetry (DSC): DSC was performed on a differential scanning calorimeter 204F1/ASC Phönix from Netzsch (Selb, Germany). Crucibles and lids made of aluminum were used. Measurements were performed in a temperature range from −20 to 250 °C using heating rates of 5, 10, 15 and 20 K∙min^−1^. As purge gas, a flow of dry nitrogen (20 mL∙min^−1^) was used. For evaluation of data, the Proteus Thermal Analysis Software (Version 5.2.1, NETZSCH-Geraetebau GmbH, Selb, Germany, 2011) and OriginPro 8G (Version 8.0951, OriginLab Corporation, Northampton, MA, USA, 2008) was used.

Rheology: In situ rheology was performed on an oscillatory plate rheometer MCR 101/SN 80753612 from Anton Paar (Graz, Austria). All measurements were performed within the linear viscoelastic regime (LVE) using a PP08 measuring system. For evaluation of data, the RheoPlus/32 software (V 3.40, Anton Paar Germany GmbH, Ostfildern, Germany, 2008) and OriginPro 8G (Version 8.0951, OriginLab Corporation, Northampton, MA, USA, 2008) were used.

Glass tube furnace: The thermal reduction of **GO-Cu(II)** was performed in a glass tube furnace (Mod. RSR-B120/750/11) from Nabertherm GmbH (Lilienthal, Germany).

Freeze drying: Freeze drying was performed on a LyoQuest freeze dryer from Telstar (Utrecht, The Netherlands) operating at −80 °C and 0.18 mbar.

Ultrasonicator: For the dispersion of the **GO**-species via ultrasonication a sonication tip Vibra Cell VCX500 from Zinsser Analytic (Frankfurt, Germany) was used.

Flame atomic absorption spectroscopy (FAAS): FAAS was performed on a novAA 350 #113A0641 Tech: Flamme spectrometer from Analytik Jena AG (Jena, Germany) using Aspect LS 1.4.1.0 (Analytik Jena AG, Jena, Germany) as software. Therefore, external calibration and calibration via doping were performed. To determine the copper-content within **TRGO-Cu_2_O** the samples were burned to ash at 800 °C under atmospheric conditions and were dispersed in nitric acid (2 M). This solution was diluted in a 1:1 ratio with a potassium chloride solution (0.2%) and a mixture containing 25 mL of the dispersed nitric sample solution and 25 mL of the potassium chloride solution.

Transmission electron microscopy (TEM): TEM investigations were performed using a EM 900 transmission electron microscope from Carl Zeiss Microscopy GmbH (Oberkochen, Germany) and the images were taken with a SSCCD SM-1k-120 camera from TRS (Moorenweis, Germany). For sample preparation, **TRGO-Cu_2_O** was dispersed in water and sprayed on a carbon-layered copper grid. After one minute, the excess solution was removed with filter paper and the samples were dried at room temperature.

X-ray diffraction (XRD): XRD-measurements were performed on a D8 X-ray diffractometer from Bruker AXS GmbH (Karlsruhe, Germany). For analysis of the raw data, Diffrac. Suite EVA 3.1 (Bruker AXS GmbH, Karlsruhe, Germany) with an integrated database for the determination of the phases was used as software. For sample preparation, **TRGO-Cu_2_O** was rubbed in the presence of isopropanol and was put on a glass slide. For evaluation of data, OriginPro 8G (Version 8.0951, OriginLab Corporation, Northampton, MA, USA, 2008) was used.

### 2.3. General Synthesis Procedure for the Preparation of Trivalent Azides

The synthesis was carried out under a dry atmosphere of nitrogen. A two-necked round-bottom flask equipped with magnetic stir bar, rubber septum and gas tap was heated under vacuum and flushed with nitrogen several times. 4-Dimethylaminopyridine (0.2 eq) was added to **2** (1.0 eq) dissolved in dry DMF and the solution was stirred for ten minutes at room temperature. Afterwards, the desired anhydride or acid chloride (6.0–8.0 eq) was added dropwise to the reaction mixture and the solution was stirred at room temperature. After finishing the reaction, the crude product was either purified by extraction or column chromatography and the obtained product was dried in high vacuo. As determined via NMR-spectroscopy, final products contain up to 25% impurities such as free epichlorohydrine and bivalent residues as trimethylolpropane triglycidyl ether was used in technical grade (see ^1^H-NMR spectrum in Supplementary Materials Figure S1).

## 3. Results

### 3.1. Synthesis of Trivalent Alkyne and Trivalent Azides

**Figure 1:**
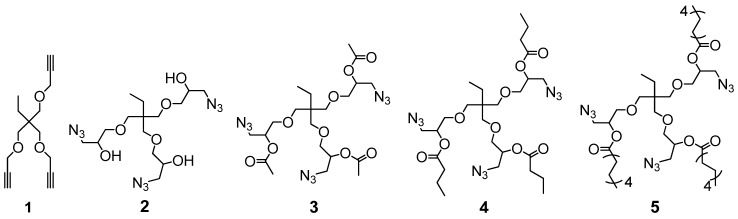
Structures of trivalent alkyne **1** and trivalent azides **2**, **3**, **4** and **5**.

In Figure 1, an overview of the synthesized trivalent alkyne **1** and the trivalent azides is given. Trivalent alkyne **1** and trivalent azide **2** have been synthesized according to literature [22,58,59].

**Scheme 1:**
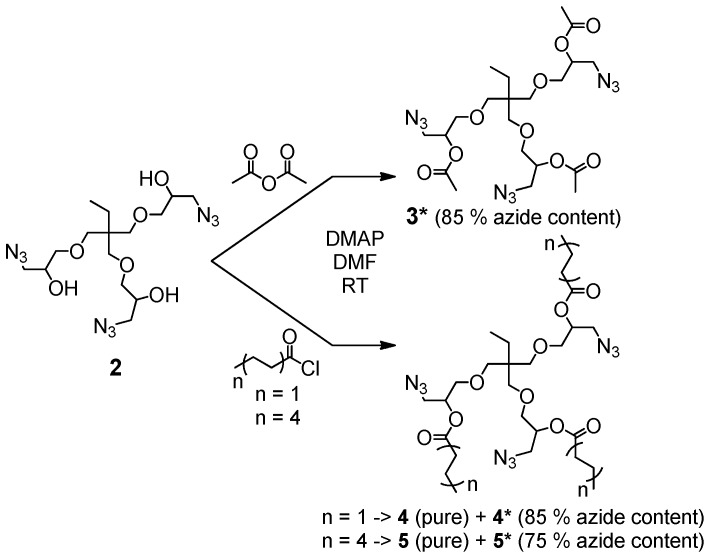
Synthesis of trivalent azides 3*, **4**, 4*, **5** and 5*.

The synthesis of the trivalent azides 3*, **4**, 4*, **5** and 5* is presented in Scheme 1, and details are given in the general synthesis procedure in Section 2.3 and in the Supplementary Materials. In brief, the trivalent azide **2** was either converted with acetic anhydride or the desired acid chloride at room temperature in dry DMF and in the presence of DMAP as a nucleophilic basic catalyst.

The trivalent azide 3* was obtained as a light-yellow, viscous liquid (85% azide content) containing the bivalent azide and free epichlorohydrine present in the starting material (trimethylolpropane triglycidyl ether (technical grade)). This mixture was further studied without purification to investigate its suitability for easy preparable and up-scalable room temperature-based self-healing nanocomposites relying on “click-crosslinking“ reactions. For the synthesis of the azides **4** and **5**, a pure trivalent compound (**4** and **5**) and a mixture of bi- and trivalent product (4* and 5*, 85 and 75% azide content, respectively) were obtained and further separated via column chromatography. All prepared multivalent azide- and alkyne-functionalized compounds were characterized via NMR- and IR-spectroscopy as well as ESI-TOF mass spectrometry proving their purity and functional group content (for more details see Supplementary Materials Figures S2–S5: ^1^H- and ^13^C-NMR spectra of trivalent azides **4** and **5**).

### 3.2. DSC Investigation of “Click-Crosslinking” Trivalent Alkyne and Trivalent Azides

Thermal analysis by differential scanning calorimetry (DSC) provides useful information about the relationship between the extent of a (crosslinking) reaction and the required time of curing at a certain temperature. Furthermore, information about kinetic parameters can be retained. Thus, DSC analysis is helpful to obtain a wide range of data of the investigated “click-crosslinking” reactions of trivalent alkyne **1** and trivalent azides 3*, **4**, 4*, **5** and 5* such as the enthalpy of the reaction (Δ*H*), the onset temperature (*T*_onset_), the temperature at the maximum of the DSC curve (*T*_p_) and the apparent activation energy of the reaction (*E*_a, app_). It should be mentioned, that the experimentally determined activation energies are indicated as apparent activation energies, since it is well known that the physical conditions during crosslinking are at least partially restricted by the mass and heat transport in the solid phase, consequently influencing the internal energy as well as the vibrational states of the investigated reactants [60,61]. Furthermore, it should be emphasized that our prepared **TRGO-Cu_2_O** catalyst is composed of several graphene sheets showing a lack in dispersibility with the reactants due to missing functional groups of increasing polarity. Thus, a relatively high apparent activation energy is expected—a phenomenon also observed in graphene oxide nanocomposite epoxy coatings [62].

**Figure 2:**
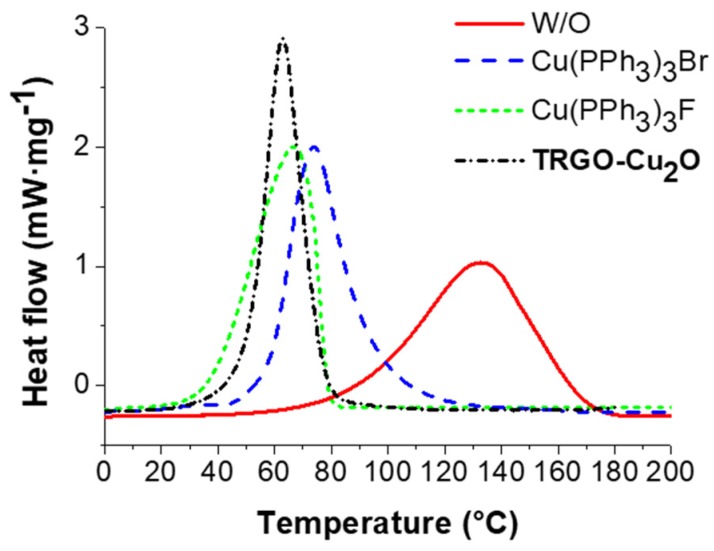
Dynamic scanning calorimetry (DSC) measurements of the “click-crosslinking” reaction of trivalent alkyne **1** and trivalent azide 3* with Cu(PPh_3_)_3_Br, Cu(PPh_3_)_3_F, Cu_2_O on graphene-oxide (**TRGO-Cu_2_O**, prepared at 600 °C) as well as without catalyst (W/O) at a heating rate of 5 K∙min^−1^.

Via DSC investigations, the “click-crosslinking” reaction conversion can be estimated with respect to a determined reference value, a maximum Δ*H* value (262 kJ∙mol^−1^) for a reference click reaction between phenylacetylene and benzyl azide when quantitatively forming one triazole unit and therefore being representative of one successful “click” reaction. This reference value is in line with reported literature values for “click” reactions ranging between 210 to 270 kJ∙mol^−1^ [63]. DSC measurements for the “click-crosslinking” reaction of trivalent alkyne **1** with trivalent azide 3* investigated at a heating rate of 5 K∙min^−1^ in the presence of different homo- and heterogenous copper(I) catalysts (1 mol % per functional group) as well as without catalyst (W/O) are plotted in Figure 2 and the obtained results are summarized in Table 1.

For the Huisgen cycloaddition of trivalent alkyne **1** and trivalent azide 3* (Table 1, Entry 1) 78% conversion was achieved corresponding to an enthalpy of 205 kJ∙mol^−1^. Crosslinking took place at high temperatures and *T*_onset_ and *T*_p_ were observed at 91 and 133 °C, respectively. In the case of the homogenous catalysts (Cu(PPh_3_)_3_F and Cu(PPh_3_)_3_Br, Table 1, Entry 2 and 3), the observed enthalpies were 191 and 185 kJ∙mol^−1^, consequently showing a conversion of 73 and 71%, respectively. By using **TRGO-Cu_2_O** as a catalyst for “click-crosslinking” **1** and 3* (Table 1, Entry 4), an enthalpy of 177 kJ∙mol^−1^ was observed corresponding to 67% conversion. Moreover, the lowest apparent activation energy (55 kJ∙mol^−1^) and the lowest maximum peak temperature *T*_p_ (63 °C) were achieved in the presence of **TRGO-Cu_2_O** and the lowest *T*_onset_ (39 °C) by using Cu(PPh_3_)_3_F. According to these results, **TRGO-Cu_2_O** and Cu(PPh_3_)_3_F were the best catalysts for the “click-crosslinking“ reaction of trivalent alkyne **1** and trivalent azide 3*. 

**Figure 3:**
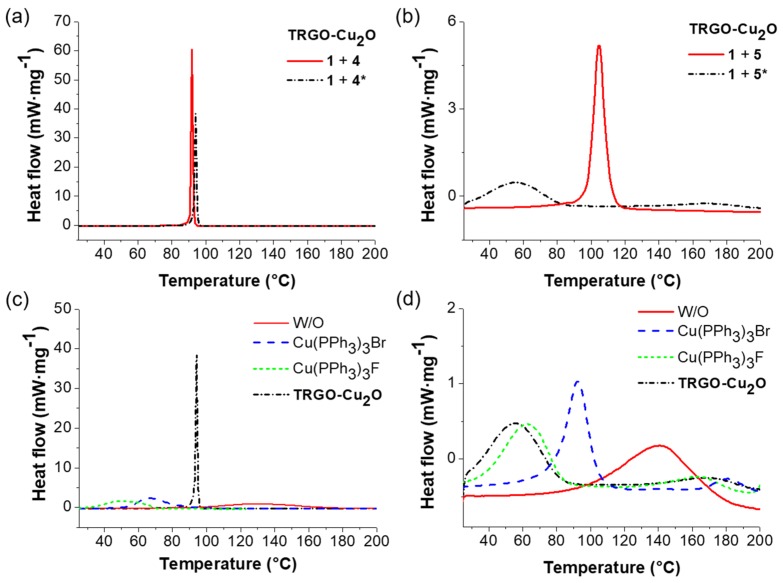
DSC measurements of the “click-crosslinking” reaction of trivalent alkyne **1** and (**a**) trivalent azides **4** (red curve) or 4* (black curve) with **TRGO-Cu_2_O**, (**b**) trivalent azides **5** (red curve) or 5* (black curve) with **TRGO-Cu_2_O** (prepared at 600 °C), (**c**) trivalent azide 4* with different catalysts as well as without catalyst (W/O), (**d**) trivalent azide 5* with different catalysts as well as without catalyst (W/O) (all at a heating rate of 5 K∙min^−1^).

To investigate the activity of the pure trivalent azides **4** and **5** in comparison to the partially functionalized trivalent azides 4* and 5* (85 and 75% azide content) together with trivalent alkyne **1** in the CuAAC crosslinking reaction, DSC measurements were run by using **TRGO-Cu_2_O** as a catalyst (1 mol % per functional group). The DSC thermograms at 5 K∙min^−1^ with **TRGO-Cu_2_O** as a catalyst are plotted in Figure 3a,b and the obtained results are summarized in Table 1.

For “click-crosslinking” trivalent alkyne **1** with trivalent azide **4** (Table 1, Entry 5), the observed enthalpy was 233 kJ∙mol^−1^, corresponding to 89% conversion. In comparison, the observed enthalpy for “click-crosslinking” trivalent alkyne **1** with trivalent azide 4* (Table 1, Entry 9) showed a slightly lower enthalpy value of 211 kJ∙mol^−1^ and a conversion of 81%, related to the presence of the bivalent byproduct consequently lowering the conversion. In contrast, the reaction temperatures (*T*_p_ and *T*_onset_) were decreased for “click-crosslinking” trivalent alkyne **1** with trivalent azide 5*, mainly attributed to a lower viscosity and therefore, to a faster diffusion. Thus, an enthalpy of 248 kJ∙mol^−1^ was observed for “click-crosslinking” trivalent alkyne **1** with trivalent azide **5** (Table 1, Entry 10) at relatively high reaction temperatures corresponding to 95% conversion. In comparison, a lower enthalpy of 127 kJ∙mol^−1^ (48% conversion) was measured for the “click-crosslinking” reaction of trivalent alkyne **1** with trivalent azide 5* (Table 1, Entry 14) while the reaction temperatures were reduced (*T*_onset_ = 32 °C, *T*_p_ = 56 °C). Consequently, further investigations towards easily up-scalable room temperature-based self-healing nanocomposites were continued by using trivalent azides 4* and 5*.

DSC thermograms of “click-crosslinking” trivalent alkyne **1** with trivalent azides 4* or 5* without catalyst (Huisgen cycloaddition) as well as with different catalysts (1 mol % per functional group) are illustrated in Figure 3c,d, respectively. For the uncatalyzed crosslinking reaction of trivalent alkyne **1** with trivalent azide 4* (Table 1, Entry 6), a high reaction enthalpy of 227 kJ∙mol^−1^ and high reaction temperatures (*T*_onset_ = 94 °C, *T*_p_ = 125 °C) were observed, corresponding to a conversion of 87%. For the homogenous catalyst Cu(PPh_3_)_3_F the lowest enthalpy was observed, while the click reaction happened at relatively low temperatures (Table 1, Entry 8). The determined apparent activation energy for the Huisgen cycloaddition was 75 kJ∙mol^−1^, while an enhanced apparent activation energy was detected for the click reaction using **TRGO-Cu_2_O** (97 kJ∙mol^−1^). In contrast, a lower apparent activation energy was determined for Cu(PPh_3_)_3_F and Cu(PPh_3_)_3_Br, showing values of 61 and 70 kJ∙mol^−1^ (Table 1, Entry 7 and 8), respectively. When reacting trivalent alkyne **1** with trivalent azide 5*, lower conversions were observed for the (uncatalyzed) Huisgen cycloaddition reaction as well as for all “click-crosslinking” reactions. Thus, a conversion of 66% was achieved for the uncatalyzed reaction (Table 1, Entry 11), corresponding to an enthalpy of 174 kJ∙mol^−1^, while “click-crosslinking” happened at high temperatures (*T*_onset_ and *T*_p_ of 102 and 141 °C). In comparison to the other catalysts, **TRGO-Cu_2_O** (Table 1, Entry 14) resulted in the lowest enthalpy of 127 kJ∙mol^−1^, which was achieved at *T*_onset_ and *T*_p_ of 32 and 56 °C, respectively. In the case of the homogeneous catalysts (Cu(PPh_3_)_3_F and Cu(PPh_3_)_3_Br) a similar conversion of 53 to 57% was observed, while lower reaction temperatures were achieved in the presence of Cu(PPh_3_)_3_F (Table 1, Entry 13, *T*_onset_ = 38 °C, *T*_p_ = 62 °C). According to the obtained results, Cu(PPh_3_)_3_F turned out to be the best catalyst for the “click-crosslinking” reaction of trivalent alkyne **1** and trivalent azides 4* or 5*.

### 3.3. DSC Investigation of “Click-Crosslinking” Trivalent Alkyne **1** and Trivalent Azides 3*, 4* and 5* at 0 °C

We were interested in quantifying “click-crosslinking” reactions during preparation and mixing of the components, thus understanding whether “click” reactions at 0 °C play an essential role. Therefore, DSC measurements were applied to investigate the kinetic behavior of the “click-crosslinking” reaction between trivalent alkyne **1** and trivalent azides 3*, 4* and 5* with different chain lengths to find a suitable and fast catalytic system for the CuAAC. As the usage of Cu(PPh_3_)_3_F resulted in high enthalpies at low crosslinking temperatures in the previously performed experiments, Cu(PPh_3_)_3_F was chosen as catalyst to check the activity of the multivalent azides and alkynes within “click-crosslinking” at 0 °C. Therefore, 1:1 mixtures of trivalent alkyne **1** and different trivalent azides (3*, 4* or 5*) together with Cu(PPh_3_)_3_F (1 mol % per functional group) were prepared. Immediately after preparation of the mixtures, DSC measurements were run at a heating rate of 5 K∙min^−1^ to observe the enthalpy of the “click-crosslinking” reaction at time zero (Δ*H*_0_). Afterwards, the mixtures were stored at 0 °C and further DSC investigations were conducted in defined time intervals and the conversion was determined (for more details see Supplementary Materials Figure S4 and Table S1).

**Figure 4:**
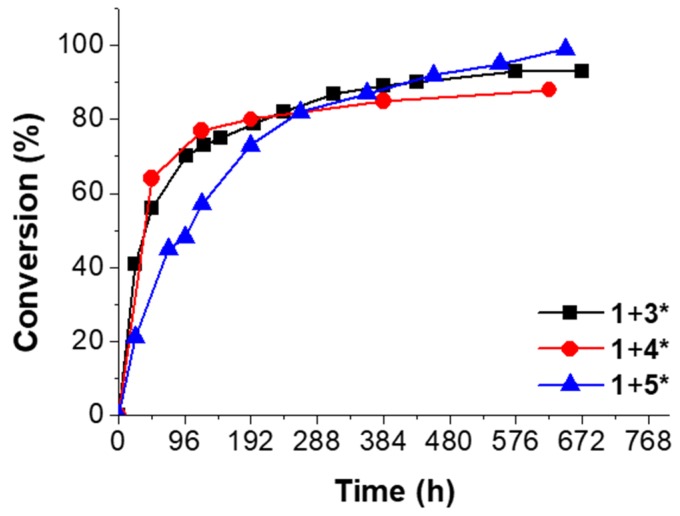
Conversion vs. time of the “click-crosslinking“ reaction of trivalent alkyne **1** and trivalent azides 3* (black squares), 4* (red circles) or 5* (blue triangles) at 0 °C with Cu(PPh_3_)_3_F as catalysts at a heating rate of 5 K∙min^−1^.

Immediately after mixing trivalent alkyne **1** and trivalent azide 3*, the measured enthalpy of the “click-crosslinking” reaction was 186 kJ∙mol^−1^, and *T*_onset_ and *T*_p_ were 39 and 67 °C, respectively (Figure 4, black squares; Supplementary Materials, Table S1, Entry 1). After 48 h storage at 0 °C, the “click-crosslinking” enthalpy decreased around one half of its initial value and reached 82 kJ∙mol^−1^, corresponding to 56% conversion (Supplementary Materials, Table S1, Entry 3). After 312 h, the “click-crosslinking” enthalpy decreased further to 24 kJ∙mol^−1^ (13 days, 87% conversion, Supplementary Materials, Table S1, Entry 9). Afterwards, the conversion did not show any significant increase, and the “click-crosslinking” reaction between alkyne **1** and azide 3* reached its maximum conversion of 93% at 0 °C after 576 h (24 days, Supplementary Materials, Table S1, Entry 12).

The mixture of trivalent alkyne **1** and trivalent azide 4* showed a high enthalpy of 222 kJ∙mol^−1^ immediately after sample preparation, and *T*_onset_ and *T*_p_ were 37 and 54 °C, respectively (Figure 4, red circles; Supplementary Materials, Table S1, Entry 14). After 48 h, the “click-crosslinking” enthalpy decreased to 80 kJ∙mol^−1^ and a conversion of 64% was observed (Supplementary Materials, Table S1, Entry 15). After 384 h, the conversion of the “click-crosslinking” reaction at 0 °C increased further to 85%, corresponding to a reaction enthalpy of 33 kJ∙mol^−1^ (Supplementary Materials, Table S1, Entry 18). In comparison, the conversion after 624 h did not significantly change and reached a constant value of 88% (27 kJ∙mol^−1^, Supplementary Materials, Table S1, Entry 19). Thus, it was concluded that the maximum “click-crosslinking” conversion obtainable by converting trivalent alkyne **1** and trivalent azide 4* at 0 °C is below 90%.

Immediately after preparation of the reaction mixture of trivalent alkyne **1** and trivalent azide 5*, a “click-crosslinking” enthalpy of 172 kJ∙mol^−1^ was observed, and *T*_onset_ and *T*_p_ were 63 and 86 °C, respectively (Figure 4, blue curve; Supplementary Materials, Table S1, Entry 20). After 96 h, the reaction enthalpy of the “click-crosslinking” reaction decreased to 89 kJ∙mol^−1^, related to a conversion of 48% (Supplementary Materials, Table S1, Entry 23). After 552 h, the enthalpy decreased further to 8 kJ∙mol^−1^ (95% conversion), and *T*_onset_ was not detectable anymore due to the very low reaction enthalpy. After 648 h, a complete “click-crosslinking” conversion was achieved, and no reaction peak was observed.

Moreover, in all crosslinking experiments investigated at 0 °C, the peak temperature *T*_p_ and the onset temperature *T*_onset_ increased in comparison to their initial values. This increase of the crosslinking temperatures is mainly attributed to the early network formation taking place at 0 °C, and is therefore related to a slowed down monomer diffusion.

To sum up the DSC investigations at 0 °C, the “click-crosslinking” reactions of trivalent alkyne **1** with trivalent azides 3* and 4* at 0 °C were faster within the first 100 h than the corresponding “click-crosslinking” reaction in the presence of trivalent azide 5*. This phenomenon was mainly attributed to the increasing chain length of the trivalent azide. Thus, under the same conditions, molecules with shorter side chains show faster “click-crosslinking” in comparison to the molecules with the longer chain length, mainly attributed to lower viscosity. Nevertheless, slightly higher conversions of the “click-crosslinking” reaction were observed on long timescales for the trivalent azides with increased chain length.

### 3.4. Rheology Investigation of “Click-Crosslinking” Trivalent Alkyne **1** and Trivalent Azides 4* and 5*

The viscoelastic and the kinetic behavior of the “click-crosslinking” reaction between trivalent alkyne **1** and trivalent azides 4* and 5* (1:1 ratio of azide and alkyne) and the resulting self-healing capability were investigated via in situ rheology. Therefore, the isothermal “click-crosslinking” reaction was directly performed on a rheometer plate at 20 °C using Cu(PPh_3_)_3_F (1 mol % per functional group) as a catalyst. The observed crossover times for the “click-crosslinking” reaction of trivalent alkyne **1** and trivalent azides 4* and 5* with Cu(PPh_3_)_3_F were 190 and 1445 minutes, respectively (see Supplementary Materials, Figure S6). By comparing these times with the crossover time of the “click-crosslinking” reaction of trivalent azide 3* with the trivalent alkyne **1** which was 35 minutes [22], it was concluded that with increasing chain length of the azide the crossover time increased. This observation was in line with the DSC investigations proving a decreased “click-crosslinking“ reactivity with increasing chain length.

### 3.5. Synthesis and Characerization of **TRGO-Cu_2_O** Prepared at Different Temperatures

**TRGO-Cu_2_O** was prepared via thermal reduction of copper(II)-modified graphene oxide in a glass tube furnace according to a previously published procedure [21,64,65], while the reduction temperature was varied between 300 to 800 °C, finally obtaining six different batches of the desired heterogeneous copper(I) catalyst (for more details see Scheme S6 in the Supplementary Materials) to optimize the synthesis procedure in terms of the catalytic activity since DSC. Investigations for “click-crosslinking“ trivalent alkyne **1** with trivalent azide 3*, 4* or 5* revealed slightly higher reaction temperatures (*T*_onset_ and *T*_p_) in comparison to the homogeneously catalyzed “click-crosslinking“ reactions in the presence of Cu(PPh_3_)_3_F.

The prepared TRGO-based copper(I) catalysts were investigated via XRD-measurements (see Supplementary Materials Figure S7a) and for all prepared **TRGO-Cu_2_O** catalysts the characteristic reflex of **GO** at 2*θ* = 11° has disappeared due to successful reduction. Furthermore, for all prepared catalysts, reflexes at 2*θ* = 38° and 2*θ* = 43° were observed related to the formed copper species (pure copper as well as copper(I)). For the **TRGO-Cu_2_O** samples prepared at 700 and 800 °C, two additional reflexes at 2*θ* = 51° and 2*θ* = 26° were detected as characteristic reflexes for pure copper and graphite, respectively. Thus, it could be concluded, that some of the oxidic groups have been eliminated during thermal reduction partially resulting in graphite-like structures. The broad signal at 2*θ* = 25° observed for all prepared TRGO-based copper(I) catalysts was caused by an interference with the sample holder. 

The prepared **TRGO-Cu_2_O** samples were further analyzed via TEM investigations (see Supplementary Materials Figure S7b–g) in which the formed nanosized copper(I) particles were visualized. The size of the particles was investigated via Image J. The particle size increased with increasing preparation temperature of **TRGO-Cu_2_O**, and average particle-diameters between 25 to 150 nm were determined. Furthermore, it was observed, that TRGO-based catalysts prepared at 700 and 800 °C displayed a more disperse distribution of nanosized copper(I) particles, which may be attributed to the formation of pure copper interacting with graphite-like structures detected in XRD investigations.

### 3.6. Crosslinking Reactions of Alkynes and Azides in the Presence of **TRGO-Cu_2_O** Prepared at Different Temperatures

The catalytic activity of the **TRGO-Cu_2_O** catalysts (prepared at different temperatures) towards “click-crosslinking” was investigated via DSC investigations. Therefore, in the first step, a model reaction between phenylacetylene and benzyl azide (1:1 ratio of azide and alkyne) was investigated at a heating rate of 5 K∙min^−1^, and the reaction temperatures (*T*_onset_ and *T*_p_), the reaction enthalpy (*ΔH*) and the conversion were recorded (for more information see Supplementary Materials Figure S8 and Table S2). While comparing the different catalysts prepared at 300 to 800 °C, it was observed that *T*_onset_ and *T*_p_ decreased with increasing temperature applied during the reduction of copper(II)-modified graphene oxide towards **TRGO-Cu_2_O**, in line with the expectation and the increasing size of the formed copper particles. Thereby, one exception was observed, and the catalyst prepared at 500 °C showed the highest peak temperature. Thus, FAAS measurements were performed to determine the loading of **TRGO** with immobilized copper nanoparticles. While most of the prepared catalysts displayed around 8 wt % of copper, a strong decrease was noted for the **TRGO-Cu_2_O** synthesized at 500 °C, directly linked to the observed reduced catalytic activity during the click reaction of phenylacetylene and benzyl azide.

In the next step, the catalytic activity was tested in a more complex system suitable for the preparation of room-temperature based self-healing epoxy nanocomposites. “Click-crosslinking“ of trivalent alkyne **1** and trivalent azide 3* (1:1 ratio of azide and alkyne, assuming 66% azide content of 3*) in the presence of different **TRGO-Cu_2_O** catalysts prepared from 300 to 800 °C (5 wt %) at a heating rate of 5 K∙min^−1^ (see Supplementary Materials Figure S10) was investigated, and the obtained reaction temperatures (*T*_onset_ and *T*_p_), the reaction enthalpies (∆*H*) and the conversions are summarized in Table 2. For comparison, the non-catalyzed reaction between trivalent alkyne **1** and trivalent azide 3* was repeated, showing similar reaction temperatures (*T*_onset_ = 96 vs. 91 °C, *T*_p_ = 130 vs. 133 °C; Table 2, Entry 1) as described before, but a reduced conversion due to the higher amount of bivalent residues (66 vs. 75% azide content, see Supplementary Materials Figure S7). All DSC measurements were performed in three independent experiments to ensure their reproducibility and to approximate the expected error. Thereby, differences especially in the peak shape were observed to be related to sample preparation and limited blending of the reactants as well as a limited diffusion to the catalyst surface being typical for a reaction directly catalyzed by a solid support [60,61].

For “click-crosslinking” trivalent alkyne **1** and trivalent azide 3*, a similar trend was observed as in the model reaction between phenylacetylene and benzyl azide. Thus, the reaction temperatures (*T*_onset_ and *T*_p_) decreased with increasing reduction temperature applied during the preparation of the different **TRGO-Cu_2_O** catalysts. Thereby, the lowest *T*_onset_ and *T*_p_ of 64 and 80 °C, respectively, were observed for “click-crosslinking” trivalent alkyne **1** and trivalent azide 3* in the presence of **TRGO-Cu_2_O** prepared at 700 °C while the catalyst prepared at 500 °C showed the worst result.

**Figure 5:**
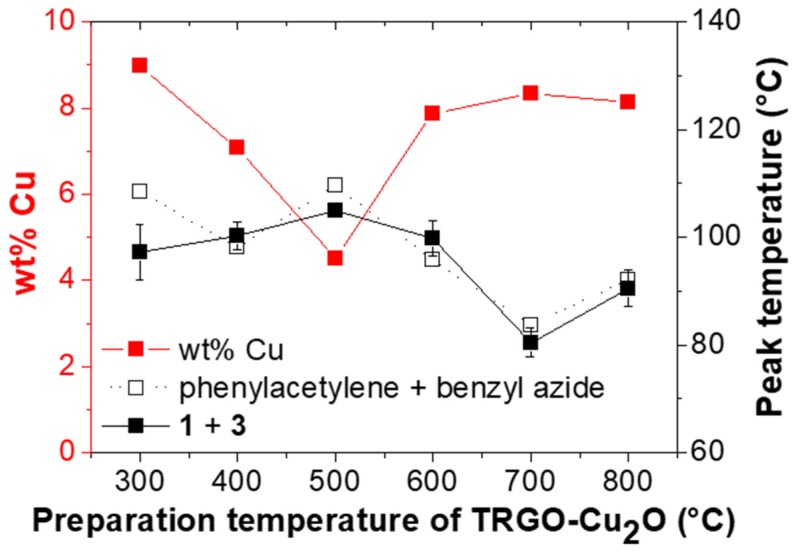
Amount (wt %) of copper within **TRGO-Cu_2_O** and peak temperature (*T*_p_) of the click reaction vs. the preparation temperature of **TRGO-Cu_2_O** for the click reaction of phenylacetylene and benzyl azide and for the “click-crosslinking” reaction of trivalent alkyne **1** and trivalent azide 3*. Please note that the lines between the measuring points are drawn to guide the eye.

The obtained peak temperatures for the click model reaction of phenylacetylene and benzyl azide as well as for the “click-crosslinking” reaction of trivalent alkyne **1** and trivalent azide 3* were correlated to the preparation temperature of **TRGO-Cu_2_O** and the corresponding amount of copper within these catalysts (see Figure 5).

The already mentioned trends within the catalytic activity of **TRGO-Cu_2_O** were well highlighted for both click reactions. The catalyst prepared at 500 °C showed a low amount of copper and consequently a high peak temperature during the click reactions, while the best results in terms of copper loading as well as peak temperature were observed for **TRGO-Cu_2_O** prepared at 700 °C. The decrease of the copper loading for the catalyst prepared at 500 °C may be related to the decrease of oxygen-functional groups with increasing reduction temperature known to act as reactive sites for the nucleation and growth of metal nanoparticles [66,67]. Following this argumentation, a further decrease in the copper loading together with a decreased catalytic activity would be expected, which was not observed in this particular study. We therefore assume that either the formation of pure copper indicated by XRD investigations is boosting the catalytic activity or that the diffusion of metal atoms, especially favored at higher temperatures, leads to the formation of non-stable metal clusters influencing the catalytic activity [68]. 

Thus, for the preparation of self-healing nanocomposites according to a previously published procedure [19], the optimized **TRGO-Cu_2_O** catalyst prepared at 700 °C was used. Thereby, the trivalent alkyne **1** together with this particular **TRGO-Cu_2_O** were directly distributed within the epoxy matrix together with µm-sized capsules filled with trivalent azide 3*. Further self-healing investigations of our optimized healing system as well as the determination of self-healing efficiencies are ongoing in our laboratories and will be part of a future publication.

## 4. Conclusions

Different low molecular weight, multivalent azides with small structural changes were synthesized and their crosslinking kinetics was investigated in a CuAAC-based curing reaction. Therefore, different homogeneous and heterogeneous copper(I) catalysts were screened and the kinetic parameters such as the reaction temperatures, the enthalpy of the reaction as well as the apparent activation energies were recorded via DSC investigations. We observed, that the “click-crosslinking” reactivity decreased with increasing chain length of the azide. Furthermore, a significant click reactivity of all investigated azides could be proven already at 0 °C.

The reaction conditions for the preparation of our home-made **TRGO-Cu_2_O** catalyst were optimized: When increasing the reaction temperature to 700 °C, the resulting copper(I) catalyst displayed the highest catalytic activity as shown in model click reactions as well as in “click-crosslinking“ reactions between trivalent alkyne **1** and trivalent azide 3*.

The tuned catalyst was subsequently dispersed in an epoxy matrix together with the trivalent alkyne **1** and the encapsulated trivalent azide 3* (µm-sized capsules). Further self-healing investigations of the so prepared capsule-based self-healing graphene-supported epoxy nanocomposites are ongoing and will be part of a future publication.

## Figures and Tables

**Table 1:** Thermal properties, reaction temperatures (*T*_onset_ and *T*_p_), reaction enthalpies (*ΔH*), apparent activation energies (*E*_a, app_) and conversions of the “click-crosslinking” reaction of trivalent alkyne **1** and trivalent azide 3*, **4**, 4*, **5** or 5* with different catalysts (1 mol %) as well as without catalyst (W/O) at a heating rate of 5 K∙min^−1^. **TRGO-Cu_2_O** was prepared at 600 °C.

Entry	Azide	Catalyst	*Mass (%)*	*T*_onset_^1^ (°C)	*T*_p_^1^ (°C)	*ΔH* ^2^ (kJ∙mol^−1^)	*E*_a, app_ (kJ∙mol^−1^)	Conversion ^3^ (%)
1	3*	W/O	-	91	133	205	83	78
2		Cu(PPh_3_)_3_Br	3.4	59	74	185	87	71
3		Cu(PPh_3_)_3_F	3.2	39	66	191	83	73
4		TRGO-Cu_2_O	7.4	51	63	177	55	67
5	4	TRGO-Cu_2_O	6.7	91	92	233	104	89
6	4*	W/O	-	94	125	227	76	87
7		Cu(PPh_3_)_3_Br	2.9	54	66	223	70	85
8		Cu(PPh_3_)_3_F	3.2	32	50	192	61	73
9		TRGO-Cu_2_O	6.9	93	94	211	97	81
10	5	TRGO-Cu_2_O	5.0	98	105	248	133	95
11	5*	W/O	-	102	141	174	100	66
12		Cu(PPh_3_)_3_Br	2.2	80	93	149	55	57
13		Cu(PPh_3_)_3_F	2.4	38	62	140	103	53
14		TRGO-Cu_2_O	5.2	32	56	127	85	48

^1^ According to our previous publication [22] and our experience, the error is typically ≈ ±5 K. ^2^ According to our previous publication [22], the error is typically ≈ ±6 kJ·mol^−1^. ^3^ Calculated with respect to the enthalpy for 100% click conversion which is *ΔH* = 262 kJ·mol^−1^ for the reference reaction of phenylacetylene and benzyl azide with 1 mol % of Cu(PPh_3_)_3_Br as catalyst.

**Table 2:** Thermal properties of the “click-crosslinking” reaction of trivalent alkyne **1** and trivalent azide 3* with **TRGO-Cu_2_O** as a catalyst (prepared at different temperatures) at a heating rate of 5 K∙min^−1^: Reaction temperatures (*T*_onset_ and *T*_p_), reaction enthalpies (*ΔH*) and conversions.

Entry	Catalyst	*T*_onset_^1^ (°C)	*T*_p_^1^ (°C)	∆*H* ^2^ (kJ⋅mol^−1^)	Conversion ^3^ (%)
1	W/O	96	130	75	29
2	**TRGO-Cu_2_O** (300 °C)	82	97	108	41
3	**TRGO-Cu_2_O** (400 °C)	80	100	101	38
4	**TRGO-Cu_2_O** (500 °C)	90	105	107	41
5	**TRGO-Cu_2_O** (600 °C)	91	100	115	44
6	**TRGO-Cu_2_O** (700 °C)	64	80	107	41
7	**TRGO-Cu_2_O** (800 °C)	72	91	113	43

^1^ Measurements were performed thrice, and the error is ≈ ±5 K. ^2^ Measurements were performed thrice and the error is ≈ ±6 kJ⋅mol^−1^. ^3^ Calculated with respect to the enthalpy for 100% click conversion, which is *ΔH* = 262 kJ⋅mol^−1^ for the reference reaction of phenylacetylene and benzyl azide with 1 mol % of Cu(PPh_3_)_3_Br as catalyst.

## Supplementary Materials

The following are available online at www.mdpi.com/2073-4360/10/1/17/s1. Scheme S1: Synthesis of trimethylolpropane tripropargyl ether (TMPTPE, **1**); Scheme S2: Synthesis of azidated trimethylolpropane tripropargyl ether (N_3_TMPTPE, **2**); Scheme S3: Synthesis of ((2-((2-acetoxy-3-azidopropoxy)methyl)-2-ethylpropane-1,3-diyl) bis (oxy))bis(3-azidopropane-1,2-diyl) diacetate **3**; Scheme S4: Synthesis of ((2-((3-azido-2-(butyryloxy)propoxy)methyl)-2-ethylpropane-1,3-diyl) bis(oxy))bis(3-azidopropane-1,2-diyl) dibutyrate **4**; Scheme S5: Synthesis of ((2-((3-azido-2-(decanoyloxy)propoxy)methyl)-2-ethylpropane-1,3-diyl) bis (oxy))bis(3-azidopropane-1,2-diyl) bis(decanoate) **5**; Scheme S6: Synthesis of modified, thermally reduced graphene oxide **TRGO-Cu_2_O**; Figure S1: ^1^H-NMR spectrum of trimethylolpropane triglycidyl ether; Figure S2: ^1^H-NMR spectrum of **4**; Figure S3: ^13^C-NMR spectrum of **4**; Figure S4: ^1^H-NMR spectrum of **5**; Figure S5: ^13^C-NMR spectrum of **5**; Figure S6: Rheological behavior of trialkyne **1** and (a) triazide **4*** or (b) triazide **5*** applying Cu(PPh_3_)_3_F as a catalyst at 20 °C; Figure S7: (a) XRD measurements of **GO-Cu(II)** and **TRGO-Cu_2_O** prepared at different temperatures (300 °C–800 °C). Reflexes of Cu, Cu_2_O, **GO** and graphite are shown for comparison. TEM images of **TRGO-Cu_2_O** prepared at (b) 300 °C, (c) 400 °C, (d) 500 °C, (e) 600 °C, (f) 700 °C and (g) 800 °C; Figure S8: DSC measurements of the click reaction of phenylacetylene and benzyl azide with **TRGO-Cu_2_O** as a catalyst at a heating rate of 5 K∙min^−1^; Figure S9: DSC measurements of the crosslinking reaction of trivalent alkyne **1** and trivalent azide **3*** without catalyst (W/O) at a heating rate of 5 K⋅min^−1^; Figure S10: DSC measurements of the “click-crosslinking” reaction of trivalent alkyne **1** and trivalent azide **3*** with **TRGO-Cu_2_O** as a catalyst prepared at (a) 300 °C, (b) 400 °C, (c) 500 °C, (d) 600 °C, (e) 700 °C and (f) 800 °C at a heating rate of 5 K⋅min^−1^; Table S1: Conversion of the “click-crosslinking“ reaction of trivalent alkyne **1** and trivalent azide **3***, **4*** or **5*** at 0 °C with Cu(PPh_3_)_3_F as a catalyst at a heating rate of 5 K∙min^−1^: Reaction temperatures (*T*_onset_ and *T*_p_), reaction enthalpies (*ΔH*) and conversions; Table S2: Thermal properties of the click reaction of phenyl acetylene and benzyl azide with **TRGO-Cu_2_O** as a catalyst (prepared at different temperatures) at a heating rate of 5 K∙min^−1^: Reaction temperatures (*T*_onset_ and *T*_p_), reaction enthalpies (*ΔH*) and conversions.
