# Supplementary material for: Improving Kinetics of “Click-Crosslinking” for Self-Healing Nanocomposites by Graphene-Supported Cu-Nanoparticles
Source: Polymers (Basel). 2017 Dec 24;10(1):17. doi: 10.3390/polym10010017 (PMC6414871; doi:10.3390/polym10010017)
Supplement: Supplementary file 1 [file polymers-10-00017-s001.docx]

Supplementary Materials

Improving Kinetics of “Click-Crosslinking” for Self-Healing Nanocomposites by Graphene-Supported Cu-Nanoparticles

Neda Kargarfard, Norman Diedrich, Harald Rupp, Diana Döhler and Wolfgang H. Binder

1. Synthesis of Trivalent Alkyne 1 and Trivalent Azides 2, 3, 4 and 5

1.1. Synthesis of 1-(Prop-2-yn-1-Yloxy)-2,2-Bis((Prop-2-yn-1-Yloxy)Methyl)Butane (Trimethylolpropane Tripropargyl Ether, TMPTPE, 1)

**Scheme S1.** Synthesis of trimethylolpropane tripropargyl ether (TMPTPE, **1**).

The synthesis of **1** was done according to literature [1,2] with slight modifications. The synthesis was carried out under a dry atmosphere of nitrogen. A three-necked round‑bottom flask equipped with mechanical stirrer, reflux condenser and rubber septum was heated under vacuum and flushed with nitrogen several times. Trimethylol propane (149.1 mmol, 20.0 g), sodium hydroxide (6.0 eq, 894.4 mmol, 35.8 g) and TBAB (…) (0.04 eq, 7.0 mmol, 2.4 g) were dissolved in distilled water (4.0 mL). Afterwards, propargyl bromide (6.0 eq, 894.4 mmol, 85.4 mL, 134.1 g, 80.0 wt % solution in toluene) was added dropwise over a period of one hour and then the temperature was increased to 60 °C and the reaction mixture was heated for 41 h at 60 °C. After the reaction has finished (TLC control, CHCl_3_, *R*_f_ = 0.85, blue stain) the reaction mixture was cooled down to room temperature. Afterwards, the solution was diluted with DCM (….) (150.0 mL) and centrifuged (three times, 10 °C, 5000 rpm, 5 min) to separate the formed salt. The combined organic phases were extracted with distilled water (five times, 500.0 mL) and dried over Na_2_SO_4_. After filtration, the solvent was removed in vacuo and the crude product was purified by column chromatography (SiO_2_, 230–400 mesh, Merck (Darmstadt, Germany), CHCl_3_, *R*_f_ = 0.62, blue stain) to obtain **1** as a light-yellow liquid in a yield of 75%.

^1^H-NMR (CDCl_3_, 400 MHz): *δ* 0.87 (t, 3H, H_1_, ^3^*J*_H,H_ = 7.6 Hz), 1.42 (q, 2H, H_2_, ^3^*J*_H,H_ = 7.6 Hz), 2.39 (t, 3H, H_3_, ^4^*J*_H,H_ = 2.4 Hz), 3.40 (s, 6H, H_4_), 4.11 (d, 6H, H_5_, ^4^*J*_H,H_ = 2.4 Hz).

^13^C‑NMR (CDCl_3_, 100 MHz): *δ* 7.5 (C_1_), 22.7 (C_2_), 42.8 (C_3_), 58.6 (C_4_), 70.3 (C_5_), 74.0 (C_6_), 80.1 (C_7_).

Electrospray ionization time of flight mass spectrometry (ESI-TOF MS) (positive mode, 0.5 mg∙mL^−1^ MeOH + NaI (20.0 mg∙mL^−1^ acetone)): *m*/*z* = 271.11 [M + Na]^+^ (simulated 271.13).

1.2. Synthesis of 3,3'-((2-((3-Azido-2-Hydroxypropoxy)Methyl)-2-Ethylpropane-1,3-Diyl)Bis(Oxy))Bis(1-Azido Propan-2-Ol) (Azidated Trimethylolpropane Triglycidyl Ether, N_3_TMPTE, 2)

**Scheme S2.** Synthesis of azidated trimethylolpropane tripropargyl ether (N_3_TMPTPE, **2**).

The synthesis of **2** was done according to literature [2,3] with slight modifications. The synthesis was carried out under a dry atmosphere of nitrogen. A three-necked round-bottom flask equipped with magnetic stir bar, rubber septum, gas tap and reflux condenser was heated under vacuum and flushed with nitrogen several times. Trimethylolpropane triglycidyl ether (technical grade, 19.8 mmol, 5.2 mL, 6.0 g) was added to the flask and was dissolved in MeOH (100.0 mL). Ammonium chloride solution (5.0 eq, 99.0 mmol, 3.5 mL, 5.3 g) and sodium azide (5.0 eq, 98.9 mmol, 6.4 g) were added to the reaction mixture, which was heated to 85 °C for 24 h. After finishing the reaction, the solvent wa85s removed under vacuo. The crude product was diluted with CHCl_3_ (150.0 mL) and extracted with water (four times, 150.0 mL), brine (one time, 80.0 mL) and water (three times, 150.0 mL). The organic phases were combined and were dried over Na_2_SO_4_. After filtration, the solvent was removed in vacuo and the product was dried in high vacuo to obtain **2** as a light-yellow, viscous liquid in a yield of 75%. The final product **2** showed 25% impurities such as free epichlorohydrine and bivalent residues.

^1^H-NMR (CDCl_3_, 400 MHz): *δ* 0.86 (m, 3H, H_1_), 1.38 (m, 2H, H_2_), 2.94 (s, 3H, H_3_), 3.48 (m, 18H, H_4_), 3.94 (m, 3H, H_5_).

^13^C-NMR (CDCl_3_, 100 MHz): *δ* 7.6 (C_1_), 23.4 (C_2_), 43.3 (C_3_), 53.3 (C_4_), 69.5 (C_5_), 72.4 (C_6_), 72.7 (C_7_).

IR (cm^−1^): *ῦ* 3399, 2961, 2873, 2097, 1446, 1277, 1102.

ESI-TOF MS (positive mode, 1.0 mg∙mL^−1^ CHCl_3_ + NaI (20 mg∙mL^−1^ acetone)): *m*/*z* = 454.31 [M − H + 2Li]^+^ (simulated 454.23).

1.3. Synthesis of ((2-((2-Acetoxy-3-Azidopropoxy)Methyl)-2-Ethylpropane-1,3-Diyl) Bis (Oxy))Bis(3-Azidopropane-1,2-Diyl) Diacetate 3

**Scheme S3.** Synthesis of ((2-((2-acetoxy-3-azidopropoxy)methyl)-2-ethylpropane-1,3-diyl) bis (oxy))bis(3-azidopropane-1,2-diyl) diacetate **3**.

The acetylation reaction of **2** was done according to literature [2] and further modified. The synthesis was carried out under a dry atmosphere of nitrogen. A two-necked round-bottom flask equipped with magnetic stir bar, rubber septum and gas tap was heated under vacuum and flushed with nitrogen several times. 4-Dimethylaminopyridine (0.2 eq, 4.7 mmol, 0.6 g) was added to **2** (23.7 mmol, 10.2 g) dissolved in dry DMF (70.0 mL) and the solution was stirred for ten minutes at room temperature. Afterwards, acetic anhydride (6.0 eq, 142.3 mmol, 13.4 mL) was added dropwise to the reaction mixture and the solution was stirred for 89 h at room temperature. After finishing the reaction, the solution was diluted with CHCl_3_ (150.0 mL) and was extracted with distilled water (three times, 200.0 mL), brine (one time, 75.0 mL) and distilled water (four times, 200.0 mL). The combined organic phases were dried over Na_2_SO_4_. After filtration, the solvent was removed in vacuo and the product was dried in high vacuo to obtain **3** as a light-yellow, viscous liquid in a yield of 75%. The final product **3** showed 15% impurities such as free epichlorohydrine and bivalent residues.

^1^H-NMR (CDCl_3_, 400 MHz): *δ* 0.82 (m, 3H, H_1_), 1.36 (m, 2H, H_2_), 2.10 (m, 9H, H_3_), 3.28 (m, 6H, H_4_), 3.45 (m, 12 H, C_5_), 5.08 (m, 3H, C_6_).

^13^C-NMR (CDCl_3_, 100 MHz): *δ* 7.6 (C_1_), 20.9 (C_2_), 22.9 (C_3_), 43.4 (C_4_), 51.0 (C_5_), 69.5 (C_6_), 71.1 (C_7_), 71.6 (C_8_), 170.1 (C_9_).

IR (cm^−1^): *ῦ* 2929, 2105, 1743, 1227, 1107.

ESI-TOF MS (positive mode, 1.0 mg∙mL^−1^ CHCl_3_ + NaI (20 mg∙mL^−1^ acetone)): *m*/*z* = 558.21 [M + H]^+^ (simulated 558.26), *m*/*z* = 580.19 [M + Na]^+^ (simulated 580.24).

1.4. Synthesis of ((2-((3-Azido-2-(Butyryloxy)Propoxy)Methyl)-2-Ethylpropane-1,3-Diyl) Bis(Oxy))Bis(3-Azidopropane-1,2-Diyl) Dibutyrate 4

**Scheme S4.** Synthesis of ((2-((3-azido-2-(butyryloxy)propoxy)methyl)-2-ethylpropane-1,3-diyl) bis(oxy))bis(3-azidopropane-1,2-diyl) dibutyrate **4**.

The synthesis was carried out under a dry atmosphere of nitrogen. A two-necked round-bottom flask equipped with magnetic stir bar, rubber septum and gas tap was heated under vacuum and flushed with nitrogen several times. 4-Dimethylaminopyridine (0.2 eq, 0.9 mmol, 0.1 g) was added to **2** (4.6 mmol, 2.0 g) dissolved in dry DMF (…) (14.0 mL) and the solution was stirred for ten minutes at room temperature. Afterwards, butyryl chloride (6.0 eq, 27.8 mmol, 2.9 mL, 3.0 g) was added dropwise to the reaction mixture and the solution was stirred for 43 h at room temperature. After finishing the reaction, the solvent was removed in vacuo and the crude product was purified by column chromatography (SiO_2_, 230–400 mesh, Merck (Darmstadt, Germany), CHCl_3_, *R*_f_ = 0.23, blue stain) to obtain **4** (pure compound) as a light-yellow liquid in a yield of 5% and **4*** as a light-yellow liquid in a yield of 70%. The final product **4*** showed 15% impurities such as free epichlorohydrine and bivalent residues.

^1^H-NMR (**4**, CDCl_3_, 400 MHz): *δ* 0.81 (m, 3H, H_1_), 0.96 (t, 6H, H_2_, ^3^*J*_H,H_ = 7.40 Hz), 1.36 (m, 2H, H_3_), 1.66 (m, 6H, H_4_), 2.32 (m, 6H, H_5_), 3.27 (m, 6H, H_6_), 3.50 (m, 12H, H_7_), 5.08 (m, 13H, H_8_).

^13^C-NMR (**4**, CDCl_3_, 100 MHz): *δ* 7.6 (C_1_), 13.6 (C_2_), 18.3 (C_3_), 22.9 (C_4_), 36.1 (C_5_), 43.4 (C_6_), 51.1 (C_7_), 69.6 (C_8_), 70.9 (C_9_), 71.6 (C_10_), 172.7 (C_11_).

IR (**4**, cm^−1^): *ῦ* 2965, 2934, 2878, 2101, 1728, 1650, 1385, 1256, 1180, 1099.

ESI-TOF MS (**4**, positive mode, 1.0 mg∙mL^−1^ CHCl_3_ + NaI (20 mg∙mL^−1^ acetone)): *m*/*z* = 664.27 [M + Na]^+^ (simulated 664.34), *m*/*z* = 756.27 [M + 3K − 2H]^+^ (simulated 756.23).

1.5. Synthesis of ((2-((3-Azido-2-(Decanoyloxy)Propoxy)Methyl)-2-Ethylpropane-1,3-Diyl) Bis (Oxy))Bis(3-Azidopropane-1,2-Diyl) Bis(Decanoate) 5

**Scheme S5.** Synthesis of ((2-((3-azido-2-(decanoyloxy)propoxy)methyl)-2-ethylpropane-1,3-diyl) bis (oxy))bis(3-azidopropane-1,2-diyl) bis(decanoate) **5**.

The synthesis was carried out under a dry atmosphere of nitrogen. A two-necked round-bottom flask equipped with magnetic stir bar, rubber septum and gas tap was heated under vacuum and flushed with nitrogen several times. 4-Dimethylaminopyridine (0.2 eq, 0.9 mmol, 0.1 g) was added to **2** (4.6 mmol, 2.0 g) dissolved in dry DMF (14.0 mL) and the solution was stirred for ten minutes at room temperature. Afterwards, decanoyl chloride (8.0 eq, 37.1 mmol, 7.7 mL, 7.1 g) was added dropwise to the reaction mixture and the solution was stirred for six days at room temperature. After finishing the reaction, the solvent was removed in vacuo and the crude product was purified by column chromatography (SiO_2_, 230–400 mesh, Merck (Darmstadt, Germany), CHCl_3_, *R*_f_ = 0.25, blue stain) to obtain **5** (pure compound) as a light-yellow liquid in a yield of 3% and **5*** as a light-yellow solid in a yield of 64%. The final product **5*** showed 25% impurities such as free epichlorohydrine and bivalent residues.

^1^H-NMR (**5**, CDCl_3_, 400 MHz): *δ* 0.81 (m, 3H, H_1_), 0.88 (t, 9H, H_2_, ^3^*J*_H,H_ = 6.86 Hz), 1.27 (m, 38H, H_3_), 1.63 (m, 6H, H_4_), 2.33 (m, 6H, H_5_), 3.27 (m, 6H, H_6_), 3.50 (m, 12H, H_7_), 5.08 (m, 3H, H_8_).

^13^C-NMR (**5**, CDCl_3_, 100 MHz): *δ* 7.6 (C_1_), 14.1(C_2_), 22.7 (C_3_), 22.9 (C_4_), 24.9 (C_5_), 29.1 (C_6_), 29.3 (C_7_), 29.4 (C_8_), 31.9 (C_9_), 34.3 (C_10_), 43.4 (C_11_), 51.1 (C_12_), 69.6 (C_13_), 70.9 (C_14_), 71.6 (C_15_), 172.9 (C_16_).

IR (**5**, cm^−1^): *ῦ* 2924, 2855, 2101, 1738, 1709, 1461, 1161, 1110.

ESI-TOF MS (**5**, positive mode, 1.0 mg∙mL^−1^ CHCl_3_ + NaI (20 mg∙mL^−1^ acetone)): *m*/*z* = 900.57 [M + Li]^+^ (simulated 900.65), *m*/*z* = 916.54 [M + Na]^+^ (simulated 916.62).

2. Synthesis of Modified, Thermally Reduced Graphene Oxide (TRGO-Cu_2_O)

**Scheme S6.** Synthesis of modified, thermally reduced graphene oxide **TRGO-Cu_2_O**.

Graphene oxide (**GO**) was prepared according to Hummer’s method [4]. The synthesis of copper(II)-modified GO (**GO-Cu(II)**) and modified, thermally reduced graphene oxide (**TRGO-Cu_2_O**) was done according to literature [5-6] and the procedure was further modified. **GO** (1.0 g) was dispersed in water (300.0 mL) *via* ultrasonication (30% amplitude) for 30 min. Copper(II) acetate hydrate (1.7 mmol, 336.0 mg) was added and the resulting mixture was again dispersed *via* ultrasonication (30% amplitude) for five minutes. The obtained suspension was stirred at room temperature overnight. After filtration the obtained solid was washed several times with water (200.0 mL) and was freeze-dried to obtain **GO-Cu(II)** (1.2 g) as a black powder. For the preparation of **TRGO-Cu_2_O** the reaction temperature was varied between 300 to 800 °C and six different batches were prepared. Therefore, the obtained **GO-Cu(II)** (300.0 mg each batch) was placed in a glass tube furnace for thermal reduction. The furnace was flushed with nitrogen (50 L⋅h^−1^) for one hour and was heated to the desired temperature (300, 400, 500, 600, 700 und 800 °C) with a heating rate of 10 K⋅min^−1^. As soon as the temperature was reached it was kept constant for 15 min under a continuous flow of nitrogen (50 L⋅h^−1^). After cooling down to room temperature the desired **TRGO-Cu_2_O** (120.0–135.0 mg each batch) was dried and obtained as a black powder.

3. NMR-Spectra

**
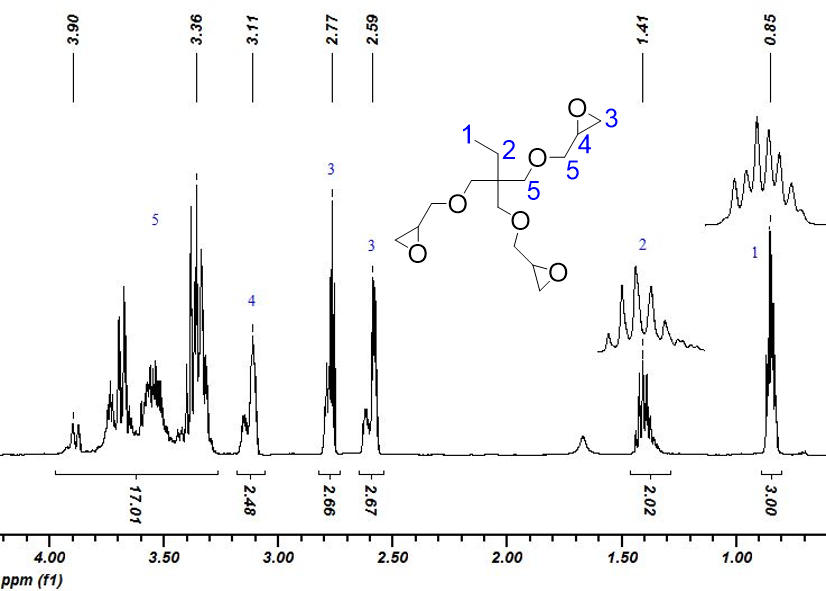
**

**Figure S1.** ^1^H-NMR spectrum of trimethylolpropane triglycidyl ether.


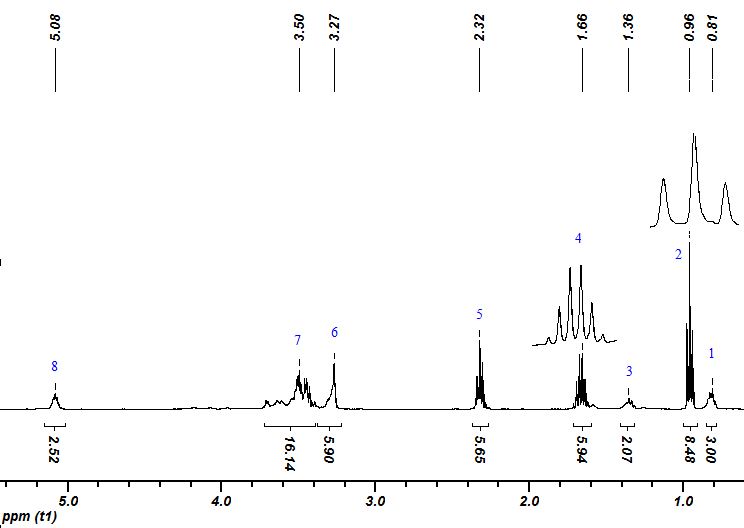


**Figure S2.** ^1^H-NMR spectrum of **4**.


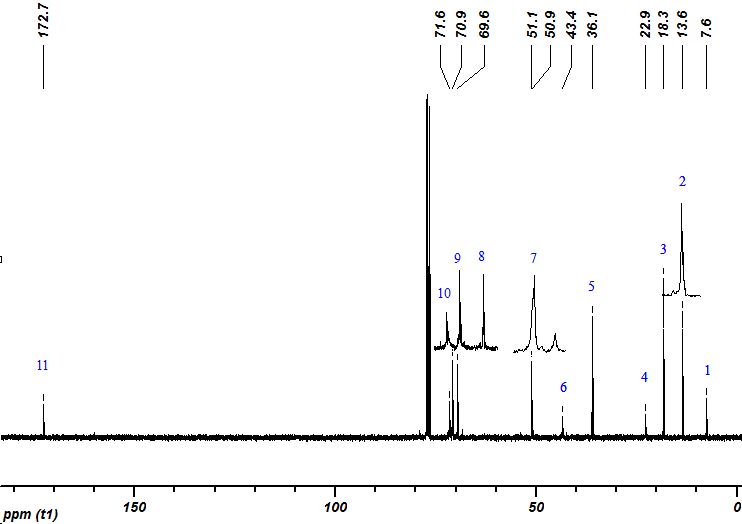


**Figure S3.** ^13^C-NMR spectrum of **4**.


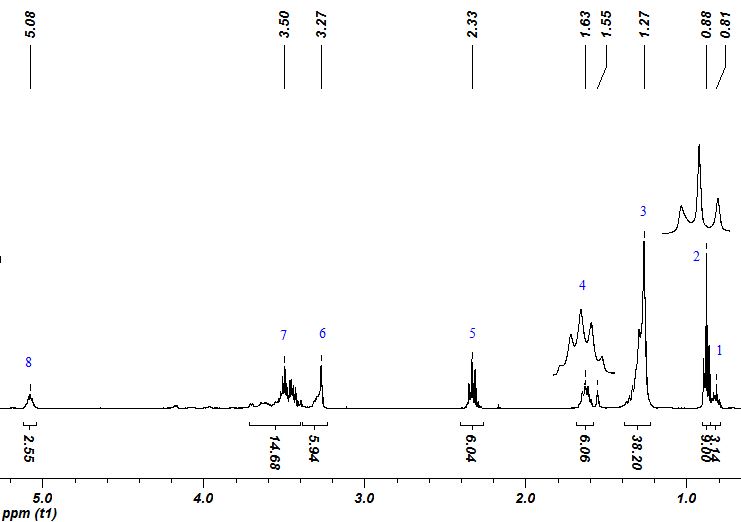


**Figure S4.** ^1^H-NMR spectrum of **5**.


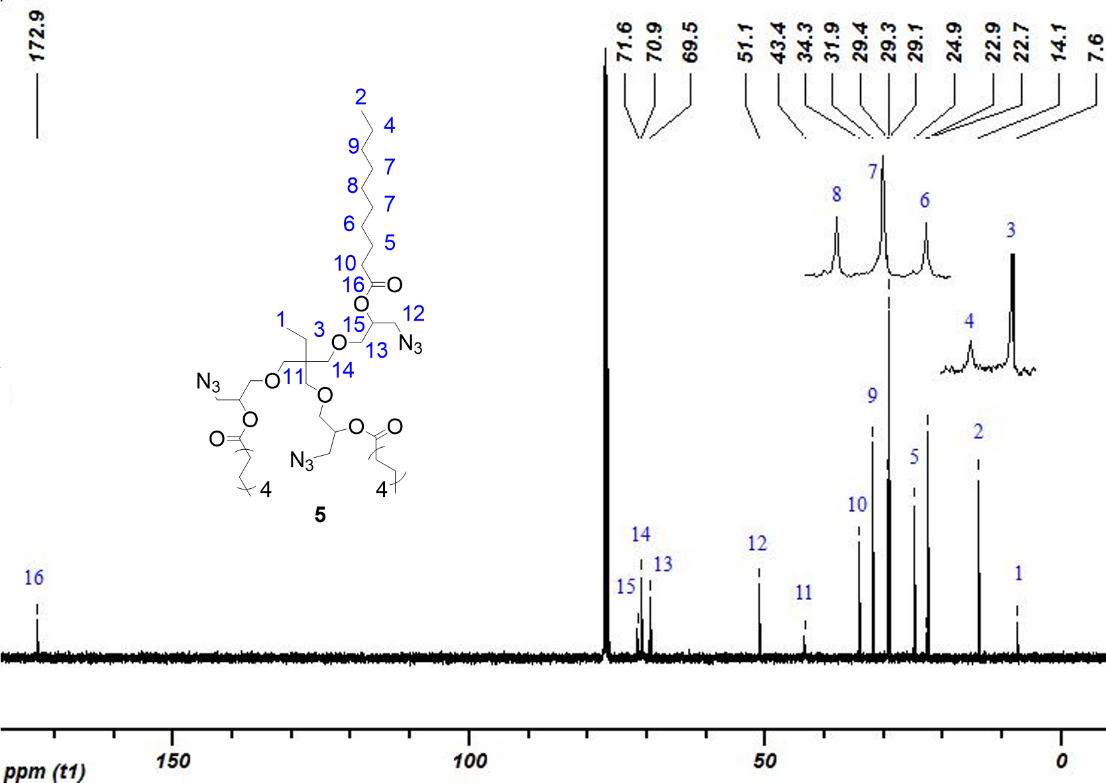


**Figure S5.** ^13^C-NMR spectrum of **5**.

4. “Click-Crosslinking” Reactions of Trivalent Alkyne 1 and Trivalent Azides 3*, 4* or 5* at 0 °C

**Table S1.** Conversion of the “click-crosslinking” reaction of trivalent alkyne **1** and trivalent azide **3**, **4*** or **5*** at 0 °C with Cu(PPh_3_)_3_F as a catalyst at a heating rate of 5 K∙min^−1^: Reaction temperatures (*T*_onset_ and *T*_p_), reaction enthalpies (*ΔH*) and conversions.

| Entry | Azide | *T*_onset_^1^ (°C) | *T*_p_^1^ (°C) | *ΔH*^2^ (kJ∙mol^−1^) | Time (h) | Conversion^3^ (%) |
| --- | --- | --- | --- | --- | --- | --- |
| 1 | 3* | 39 | 67 | 186 | 0 | 0 |
| 2 |  | 43 | 68 | 110 | 24 | 41 |
| 3 |  | 44 | 67 | 82 | 48 | 56 |
| 4 |  | 46 | 67 | 56 | 98 | 70 |
| 5 |  | 46 | 66 | 51 | 124 | 73 |
| 6 |  | 48 | 66 | 47 | 148 | 75 |
| 7 |  | 48 | 66 | 39 | 196 | 79 |
| 8 |  | 49 | 65 | 33 | 240 | 82 |
| 9 |  | 48 | 63 | 24 | 312 | 87 |
| 10 |  | 48 | 63 | 20 | 384 | 89 |
| 11 |  | 47 | 63 | 19 | 432 | 90 |
| 12 |  | 47 | 62 | 14 | 576 | 93 |
| 13 |  | 48 | 63 | 14 | 672 | 93 |
| 14 | 4* | 37 | 54 | 222 | 0 | 0 |
| 15 |  | 40 | 57 | 80 | 48 | 64 |
| 16 |  | 41 | 59 | 51 | 120 | 77 |
| 17 |  | 42 | 60 | 44 | 192 | 80 |
| 18 |  | 43 | 61 | 33 | 384 | 85 |
| 19 |  | 45 | 62 | 27 | 624 | 88 |
| 20 | 5* | 63 | 86 | 172 | 0 | 0 |
| 21 |  | 61 | 84 | 135 | 24 | 21 |
| 22 |  | 63 | 87 | 95 | 72 | 45 |
| 23 |  | 67 | 90 | 89 | 96 | 48 |
| 24 |  | 68 | 89 | 74 | 120 | 57 |
| 25 |  | 73 | 94 | 46 | 192 | 73 |
| 26 |  | 76 | 95 | 30 | 264 | 82 |
| 27 |  | 79 | 98 | 22 | 360 | 87 |
| 28 |  | 95 | 99 | 14 | 456 | 92 |
| 29 |  | - ^2^ | 101 | 8 | 552 | 95 |
| 30 |  | - ^2^ | - ^2^ | 0 | 648 | >99 |

^1^ According to our previous publication [2] and our experience the error is typically ≈ ±5 K. ^2^ According to our previous publication and our experience the error is typically ≈ ±6 kJ∙mol^−1^. ^3^ ([1 − (*ΔH*/*ΔH*^0^)] × 100), *ΔH*^0^ = 186.4 kJ∙mol^−1^, ^4^ Not detectable.

5. Rheology Investigation of “Click-Crosslinking” Trivalent Alkyne 1 and Trivalent Azides 4* and 5*


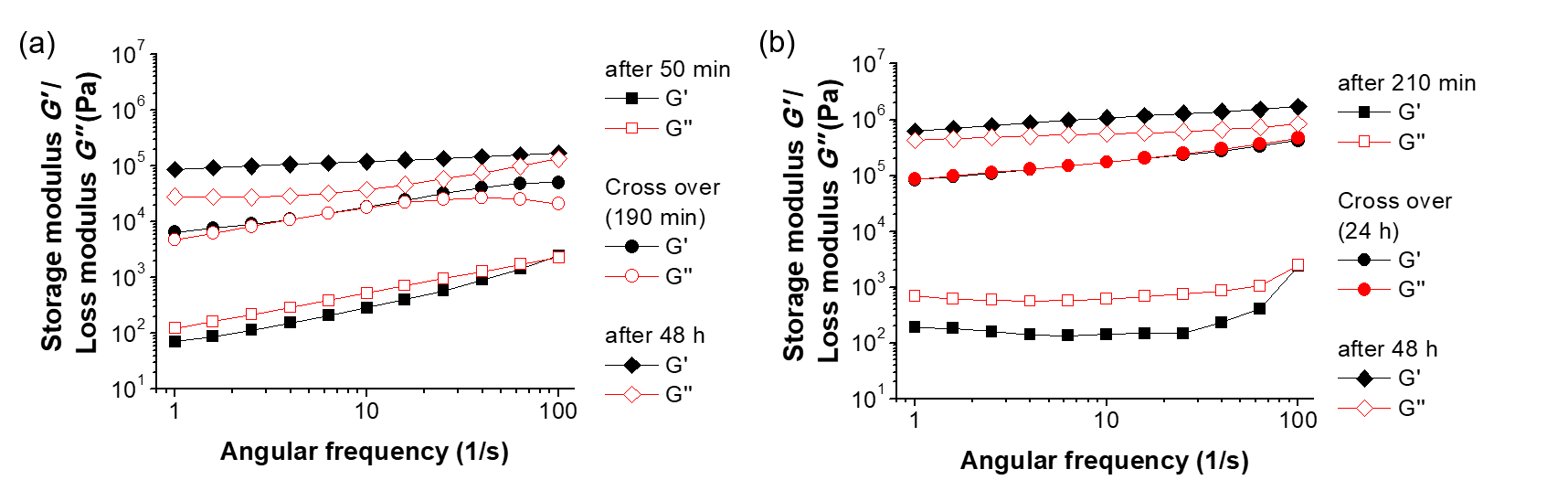


**Figure S6.** Rheological behavior of trialkyne **1** and (**a**) triazide **4*** or (**b**) triazide **5*** applying Cu(PPh_3_)_3_F as a catalyst at 20 °C.

6. Characterization of TRGO-Cu_2_O Prepared at Different Temperatures

**
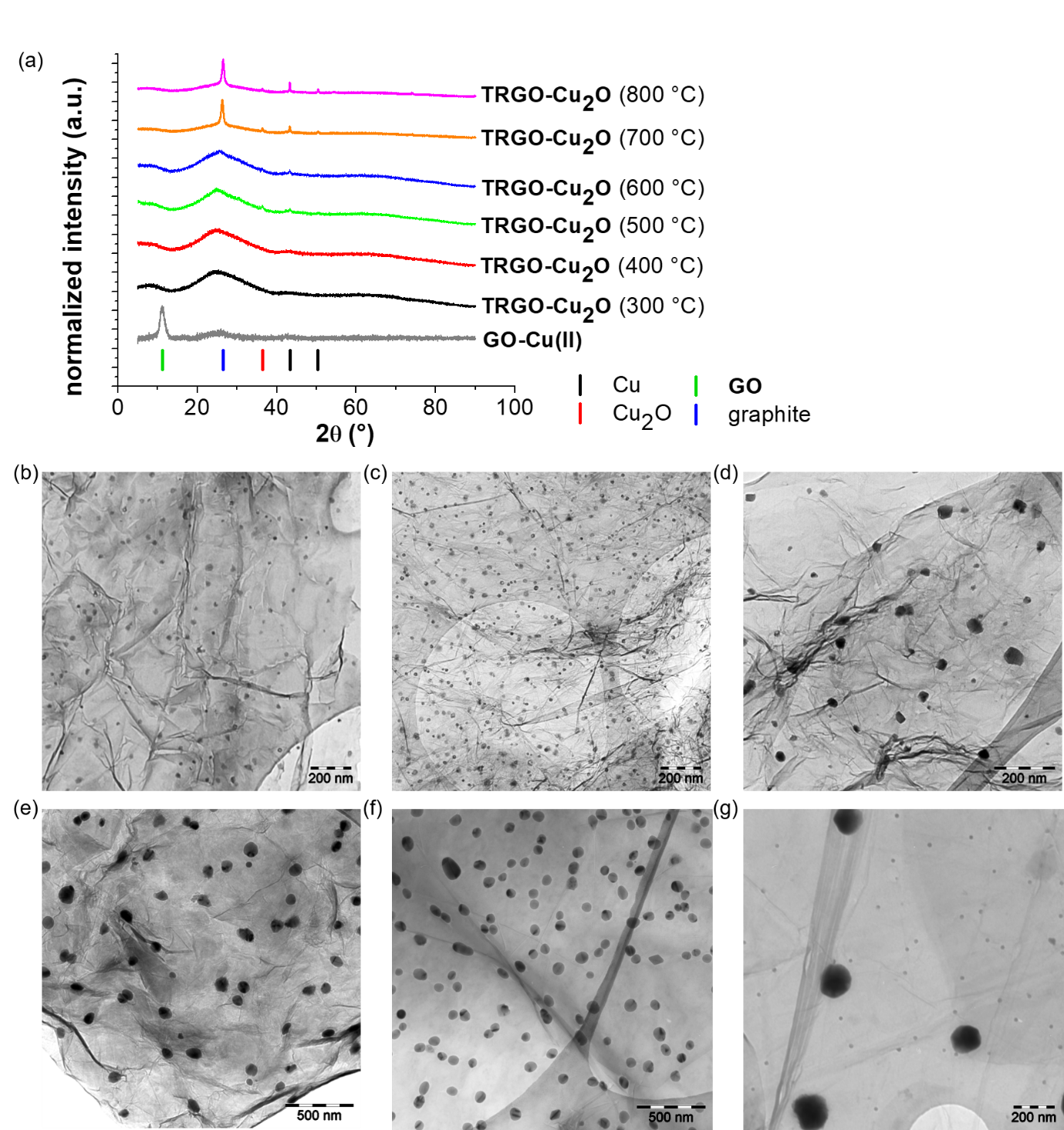
**

**
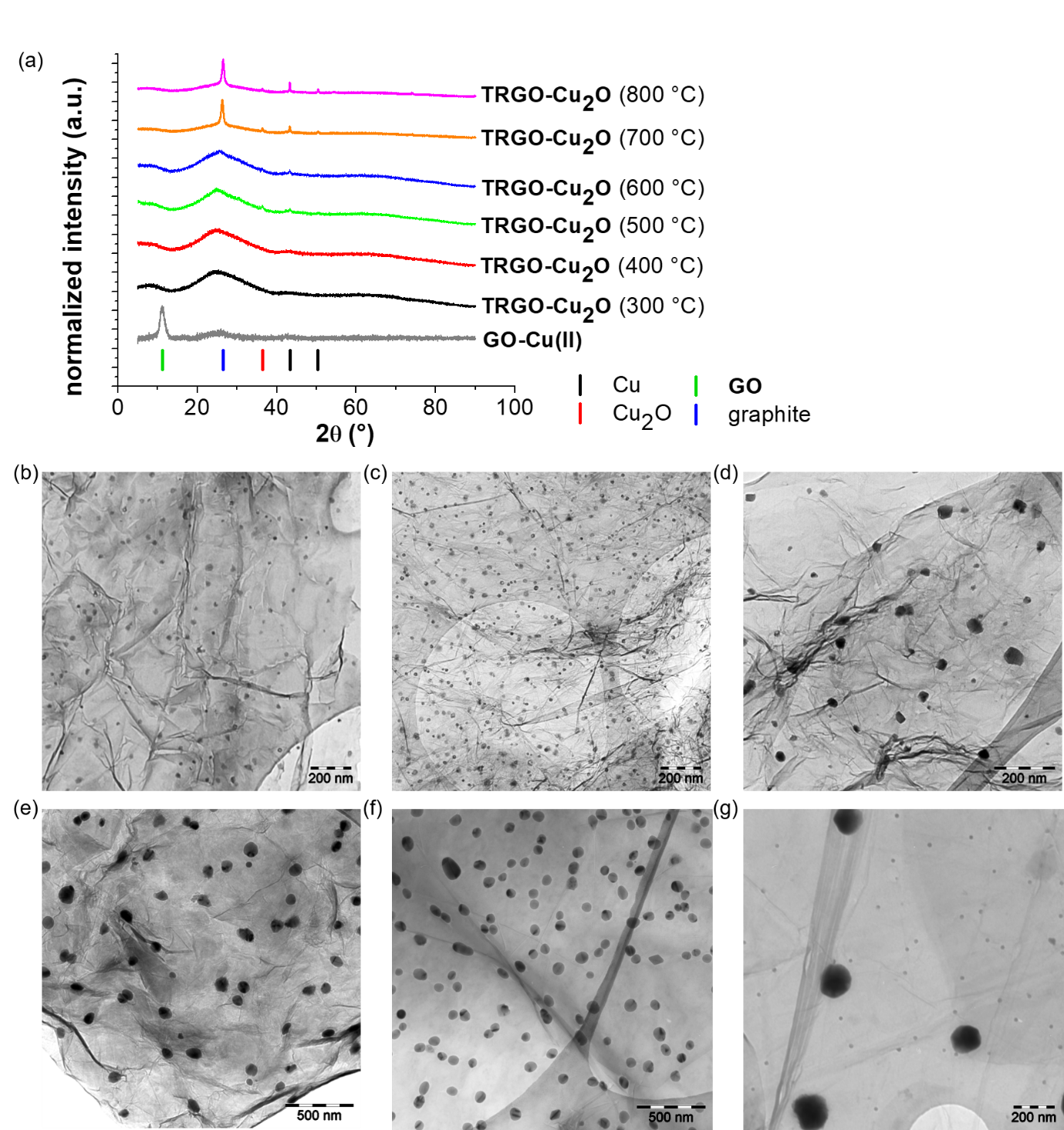
**

**Figure S7.** (**a**) XRD measurements of **GO-Cu(II)** and of **TRGO-Cu_2_O** prepared at different temperatures (300 °C–800 °C). Reflexes of Cu, Cu_2_O, **GO** and graphite are shown for comparison. TEM images of **TRGO-Cu_2_O** prepared at (**b**) 300 °C, (**c**) 400 °C, (**d**) 500 °C, (**e**) 600 °C, (**f**) 700 °C and (**g**) 800 °C.

7. Click Reactions of Alkynes and Azides in the Presence of TRGO-Cu_2_O Prepared at Different Temperatures

**
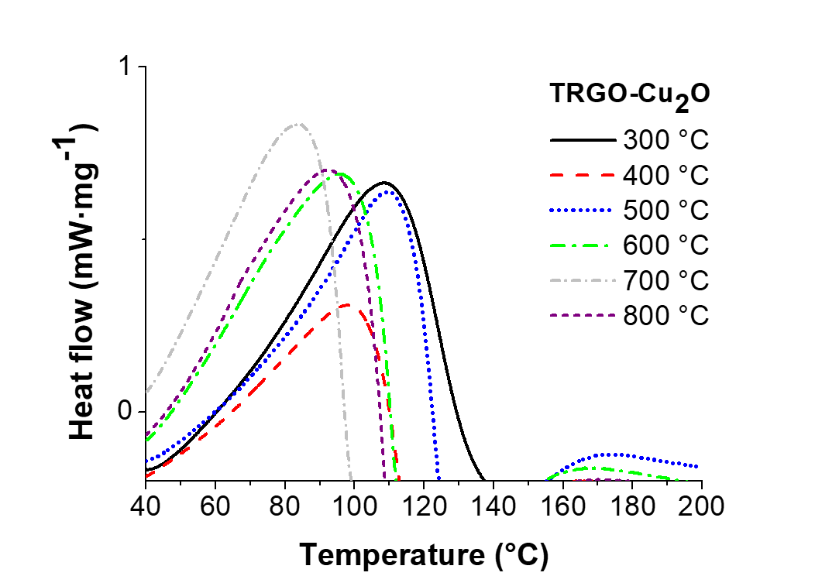
**

**Figure S8.** DSC measurements of the click reaction of phenylacetylene and benzyl azide with **TRGO-Cu_2_O** as a catalyst (prepared at different temperatures) at a heating rate of 5 K∙min^−1^.

**Table S2.** Thermal properties of the click reaction of phenylacetylene and benzyl azide with **TRGO-Cu_2_O** as a catalyst (prepared at different temperatures) at a heating rate of 5 K∙min^−1^: Reaction temperatures (*T*_onset_ and *T*_p_), reaction enthalpies (Δ*H*) and conversions.

| Entry | Catalyst | *T*_onset_^1^ (°C) | *T*_P_^1^ (°C) | ∆*H*^2^ (kJ⋅mol^−1^) | Conversion^3^ (%) |
| --- | --- | --- | --- | --- | --- |
| **1** | W/O | -^4^ | - | - | - |
| **2** | **TRGO-Cu_2_O** (300 °C) | 49 | 109 | 62 | 24 |
| **3** | **TRGO-Cu_2_O** (400 °C) | 37 | 98 | 38 | 15 |
| **4** | **TRGO-Cu_2_O** (500 °C) | 39 | 110 | 50 | 19 |
| **5** | **TRGO-Cu_2_O** (600 °C) | <37 | 96 | 47 | 18 |
| **6** | **TRGO-Cu_2_O** (700 °C) | <37 | 84 | 49 | 19 |
| **7** | **TRGO-Cu_2_O** (800 °C) | <37 | 92 | 54 | 21 |

^1^ According to our previous publication [2] and our experience the error is typically ≈ ±5 K. ^2^ According to our previous publication and our experience the error is typically ≈ ±6 kJ∙mol^−1^. ^3^ Calculated with respect to the enthalpy for 100% click conversion which is *ΔH* = 262 kJ⋅mol^−1^ for the reference click reaction of phenylacetylene and benzyl azide with 1 mol % of Cu(PPh_3_)_3_Br as catalyst. ^4^Before the Huisgen-reaction could take place, phenylacetylene evaporated already at ca. 140 °C.

**
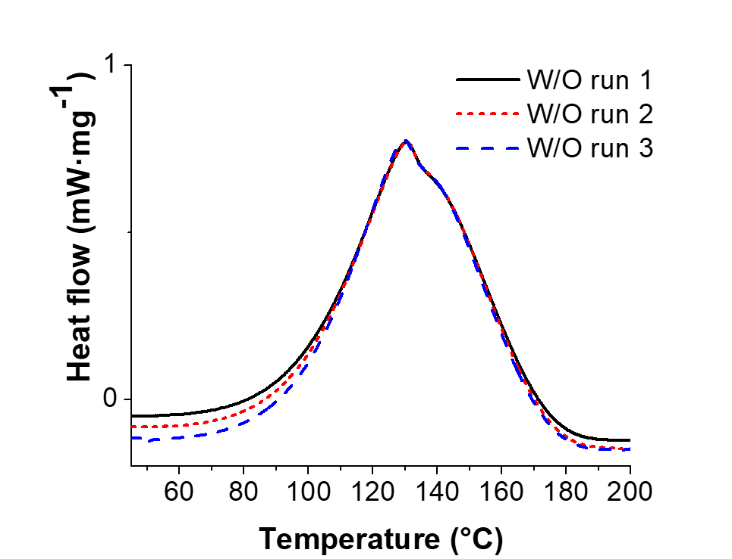
**

**Figure S9.** DSC measurements of the crosslinking reaction of trivalent alkyne **1** and trivalent azide **3** without catalyst (W/O) at a heating rate of 5 K⋅min^−1^.

**
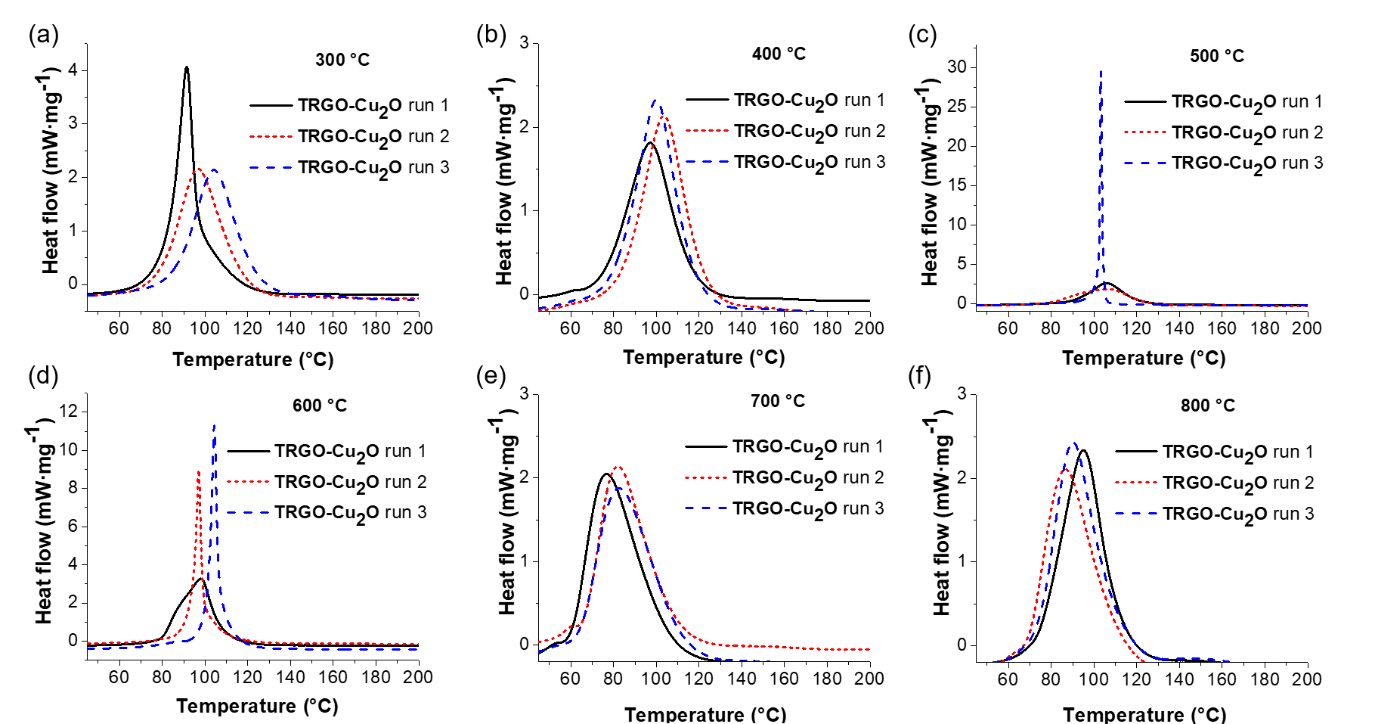
**

**Figure S10.** DSC measurements of the “click-crosslinking” reaction of trivalent alkyne **1** and trivalent azide **3** with **TRGO-Cu_2_O** as a catalyst prepared at (**a**) 300 °C, (**b**) 400 °C, (**c**) 500 °C, (**d**) 600 °C, (**e**) 700 °C and (**f**) 800 °C at a heating rate of 5 K⋅min^−1^.

© 2017 by the authors; licensee MDPI, Basel, Switzerland. This article is an open access article distributed under the terms and conditions of the Creative Commons Attribution (CC BY) license (http://creativecommons.org/licenses/by/4.0/).
